# Clinical Significance of Neuropeptide Y Expression in Pelvic Tissue in Patients with Pelvic Floor Dysfunction

**DOI:** 10.1515/biol-2019-0014

**Published:** 2019-07-30

**Authors:** Limin Zhang, Xin Luo

**Affiliations:** 1 Department of Obstetrics and Gynecology, First Affiliated Hospital of Jinan University, Jinan 510632 PR China; 2 Department of Obstetrics and Gynecology, First Affiliated Hospital of Fujian Medical University, Fuzhou 350005 PR China

**Keywords:** neuropeptide Y, pelvic floor dysfunction, pelvic organ prolapse, stress urinary incontinence

## Abstract

**Objectives:**

To investigate the neuropeptide Y (NPY) expression in the tissue of pelvic floor ligament and anterior vaginal wall in female patients with pelvic organ prolapse (POP) and stress urinary incontinence (SUI).

**Method:**

Seventeen patients with POP, 6 with SUI, 13 with POP and SUI (POP&SUI), and 10 controls were included in this study from First Affiliated Hospital of JiNan University. Immunohistochemical assay was used to examine NPY expression in the tissue of round ligament, cardinal ligament of uterus, uterosacral ligament, and anterior vaginal wall. NPY expression were compared between POP, SUI, POP&SUI and controls.

**Results:**

NPY was positive expressed in the round ligament, cardinal ligament of uterus, uterosacral ligament, and anterior vaginal subepithelial connective tissue. Compared with the control group, NPY expression in the round, cardinal, and uterosacral ligaments in patients with POP&SUI group was decreased with significant statistical difference (p<0.05). NPY expression in anterior vaginal wall was significantly decreased in POP, SUI, and POP&SUI groups compared to normal group (p<0.05). Compared to POP group, NPY expression in SUI and POP&SUI groups were significantly decreased (p<0.05), however the difference was not statistical different between SUI and POP&SUI groups (p>0.05). In POP and POP&SUI groups, the NPY expression in the cardinal ligament of uterus, uterosacral ligament, and anterior vaginal wall were negatively correlated with age (p<0.05), however, was not correlated with number of pregnancy, number of delivery, and BMI (p>0.05).

**Conclusions:**

NPY expression was reduced in the round ligament, cardinal ligament of uterus, Uterosacral ligament, and vaginal anterior wall of the patients with POP and SUI. The decreased NPY expression may play an important role in the development of pelvic floordysfunction.

## 1 Introduction

Female pelvic floor dysfunction (PFD) is the weakening of the pelvic floor support tissue caused by various reasons, thereby leading to pelvic organ displacement [1, 2]. With the increase of age, the incidence increased significantly [3, 4]. It had been reported that approximately tens of million individuals worldwide suffered from PFD, mainly including pelvic organ prolapse (POP), stress urinary incontinence (SUI), fecal incontinence (FI), and postpartum sexual dysfunction [5].

Pelvic floor nerve damage is one of the most important PFD pathogenesis. Many studies have shown that partial denervation occur in the pelvic floor muscles of patients with PFD [6]. Studies on neuromuscular pathology have revealed the morphological change in the levator ani muscle and the relationship between the distribution of vaginal mucosal nerve endings and POP and SUI [7]. Parks and his colleges [8] studied the levator ani muscle biopsy specimens of patients with PFD. Although the degree of lesion is different in each case, pathological changes are similar. Diffused neurogenic atrophy and homogenous fiber aggregation can be observed in the specimens. According to the pelvic floor nerve damage mechanism, the changes in pelvic floor neurotransmitters may also be involved in SUI pathogenesis. In the past 30 years, several studies have demonstrated the importance of neuropeptides in the development of pelvic floor dysfunction. In our present work, we investigate the NPY expression in pelvic floor ligament and anterior vaginal epithelium in female patients with POP and SUI in order to further evaluate their correlations.

## 2 Material and Methods

### 2.1 Patient

A total of 46 patients recruited from the Department of Obstetrics and Gynecology, the First Affiliated Hospital of Jinan University were included and divided into 4 groups, as follows: POP (17 cases), SUI (6 cases), POP&SUI (13 cases), and control groups (10 cases). All then include subjects had no previous vaginal and urethral surgery history or genital and urinary tract infections. The participants did not take hormones three months before surgery, and all the included cases didn’t have estrogen-related diseases, such as endometriosis and functional ovarian tumors. The control groups included cervical intraepithelial neoplasia III or carcinoma in situ or postmenopausal ovarian tumor patients.

**Informed consent**: Informed consent has been obtained from all individuals included in this study.

**Ethical approval**: The research related to human use has been complied with all the relevant national regulations, institutional policies and in accordance the tenets of the Helsinki Declaration, and has been approved by First Affiliated Hospital of Jinan University’s institutional review board.

### 2.2 Tissue extraction

In the gynecological laparotomy or vaginal hysterectomy and urinary incontinence surgery, tissue extraction was performed by senior gynecologists. The round ligament was obtained from the truncated segment of round ligament near the uterus. The cardinal ligament of uterus was obtained at a distance of 1.0 cm below the uterine artery and near the uterus. The vaginal wall was obtained at the upper and middle layers of the vaginal anterior wall. Hysiological saline was used to clean the blood on the surface; it was placed in 10% formalin solution to fix and set in an automatic tissue dehydrator for conventional dehydration. After entering in 75%, 85%, 95%, and 100% alcohol in sequence, xylene became transparent; after being immersed in wax, it was embedded into a wax block for use.

### 2.3 Immunohistochemistry assay

Conventional wax block sections were fixed on polylysine-treated anti-offset slides. Then, dewaxing and hydration were carried out. The 0.01 M sodium citrate buffer (pH of 6.0) was heated in a microwave oven, and then tissue sections were placed in it after boiling. Antigen retrieval was repeated twice at intervals of 5–10 min. Primary antibody was added in a dropwise manner overnight and then reheated for 45 min at 37 °C. Afterward, biotinylated secondary antibody was added in a dropwise manner for 20 min at 20 °C to 37 °C. Then, 100 μl of freshly prepared DAB color solution was added to each section and incubated for 5–10 min at room temperature.

### 2.4 Statistical analysis

SPSS 16.0 statistical software (SPSS, Inc., Chicago, IL, USA) was used to analyze the data. The results of measurement data was expressed by $\bar{x}\pm s.$The compassion was analyzed by one-way ANOVA. The grading data (NPY expression intensity) was analyzed by Kruskal-Wallis H test and Mann-whitney U Test. Multivariate linear regression was used for multivariate correlation analysis. Two tails p< 0.05 was statistical significance.

## 3 Results

### 3.1 General characteristics of patients

The mean age, number of pregnancy number of delivery and BMI were not statistical different in POP, SUI, POP&SUI and control groups (p>0.05), Table 1.

**Table 1:** General characteristics of the included patients

Group	No.	Age(year)	Number of pregnancy	Number of delivery	BMI(kg/m^2^)
POP	17	65.35±8.67	3.47±1.51	3.18±1.67	21.86±2.53
SUI	6	54.83±14.19	2.67±0.82	2.50±0.84	20.63±2.64
POP＆SUI	13	66.85±12.50	3.62±2.02	3.54±2.07	23.10±2.38
Control	10	59.90±13.52	2.80±1.14	2.50±1.35	21.78±1.20
		F=1.908	F=0.925	F=0.994	F=1.792
		P=0.143	P=0.437	P=0.405	P=0.163

### 3.2 Microstructure of pelvic floor

**Figure 1:**
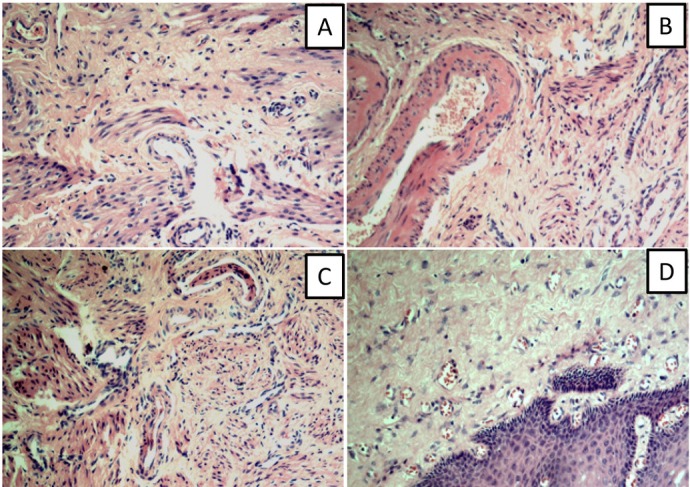
HE stain of the pelvic ligaments (A: round ligament; B: cardinal ligament of uterus; C: uterosacral ligament; D: anterior vaginal wall) ×100

Under the microscope, the patients’ round, cardinal, and uterosacral ligaments were mainly composed of connective tissue and smooth muscle containing blood vessels and nerves. The histological features of the round, cardinal, and uterosacral ligaments were similar (Figure 1 A-C). The vaginal wall consisted of the mucosal layer, muscular layer, and the outer membrane. The lamina propria of the vaginal wall mainly consists of connective tissues, which contain structures such as smooth muscle, blood vessels, and small nerve tissue (Figure. 1D).

### 3.3 Distribution of NPY in pelvic floor tissue

**Figure 2:**
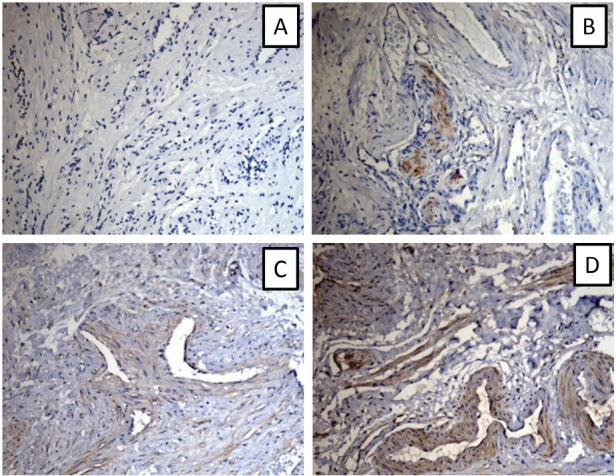
NPY expression in the pelvic ligaments (A:-; B:+; C++; D:+++) ×100

**Figure 3:**
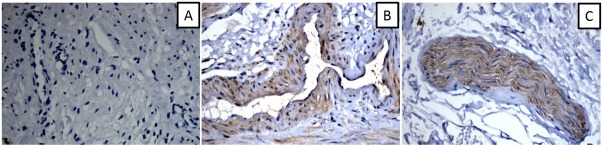
The expression of NPY around the blood vessels (A: NPY negative expression around the vessels; B: NPY positive expression around the vessels; C: NPY positive expression in the nerve fibers) ×200

**Figure 4:**
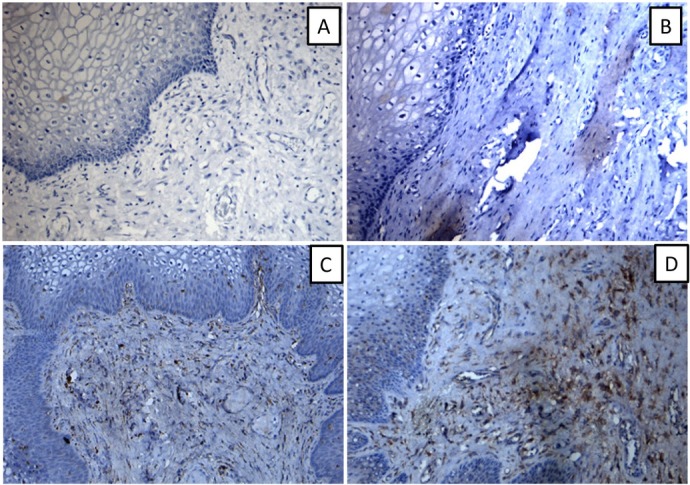
NPY expression in the anterior wall of the vagina（×100）(A:-; B:+; C++; D:+++) ×100

NPY-positive nerve fiber expression was observed in the round ligament, cardinal ligament of uterus, and uterosacral ligament (Figure. 2), which was mainly distributed around blood vessels, smooth muscles, and their surroundings (Figure. 3). NPY-stained nerve fibers were observed in the connective tissue of the vaginal mucosa, which was mainly distributed around the blood vessels (Figure. 4). NPY expression in different tissues of the patients in each group is shown in Table 2.

**Table 2:** NPY expression in different groups

Tissue	POP (n=17)	SUI (n=6)	POP&SUI (n=13)	Control(n=10)
Round ligament				
(-)	12	1	13	5
(﹢)	3	3	0	2
(++)	2	2	0	2
(+++)	0	0	0	1
Cardinal ligament of uterus				
(-)	12	1	11	4
(﹢)	4	0	2	2
(++)	1	4	0	3
(+++)	0	1	0	1
Uterosacral ligament				
(-)	13	2	13	2
(﹢)	3	1	0	3
(++)	1	2	0	3
(+++)	0	1	0	2
Connective tissue of the vaginal mucosa				
(-)	8	3	12	1
(﹢)	4	3	1	2
(++)	4	0	0	4
(+++)	1	0	0	3

Compared with the control group, NPY expression in the round, cardinal, and uterosacral ligaments in patients with POP&SUI group was decreased with significant statistical difference (p<0.05). NPY expression in anterior vaginal wall was significant decreased in POP, SUI, and POP&SUI groups compared to normal group (p<0.05). Compared to POP group, NPY expression in SUI and POP&SUI groups were significantly decreased (p<0.05), however the difference was not statistical different between SUI and POP&SUI groups (p>0.05), Table 3.

**Table 3:** NPY expression compassion among different groups in different tissue

Groups	Round ligament	Cardinal ligament of uterus	Uterosacral ligament	Connective tissue of the vaginal mucosa
POP vs SUI	P=0.045	P=0.026	P=0.031	P=0.046
POP vs POP&SUI	P=0.036	P=0.026	P=0.036	P=0.019
POP vs Control	P=0.057	P=0.453	P=0.221	P=0.039
SUI vs POP&SUI	P=0.003	P=0.001	P=0.000	P=0.188
SUI vs Control	P=0.897	P=0.406	P=0.458	P=0.005
POP&SUI vs Control	P=0.002	P=0.016	P=0.005	P=0.000

### 3.4 Correlation between NPY expression and PFD

In POP and POP&SUI groups, the NPY expression in the cardinal ligament of uterus, uterosacral ligament, and anterior vaginal wall were negatively correlated with age(p<0.05) and had no correlation with number of pregnancy, number of delivery, and BMI (p>0.05),(Table 4).

**Table 4:** Correlation between NPY expression and PFD[r, (p value)]

Group	Tissue	Age	Number of pregnancy	Number of delivery	BMI(kg/m2)
POP	Round ligament	-0.225(0.421)	-0.347(0.089)	-0.309(0.149)	-0.106(0.455)
	Cardinal ligament of uterus	-0.490(0.047)*	-0.215(0.080)	-0.177(0.400)	-0.239(0.558)
	Uterosacral ligament	-0.512(0.040)*	-0.155(0.588)	-0.695(0.192)	-0.168(0.130)
	Connective tissue of the vaginal mucosa	-0.817(0.021)*	-0.279(0.232)	-0.517(0.233)	-0.059(0.212)
SUI	Round ligament	-0.287(0.365)	-0.193(0.405)	-0.227(0.476)	-0.165(0.132)
	Cardinal ligament of uterus	-0.179(0.442)	-0.207(0.335)	-0.413(0.123)	-0.117(0.332)
	Uterosacral ligament	-0.233(0.356)	-0.236(0.385)	-0.397(0.346)	-0.231(0.446)
	Connective tissue of the vaginal mucosa	-0.326(0.652)	-0.369(0.452)	-0.298(0.267)	-0.245(0.432)
POP&SUI	Round ligament	-0.290(0.650)	-0.330(0.197)	-0.306(0.252)	-0.132(0.365)
	Cardinal ligament of uterus	-0.484(0.044)*	-0.206(0.252)	-0.532(0.512)	-0.212(0.310)
	Uterosacral ligament	-0.466(0.032)*	-0.357(0.170)	-0.326(0.129)	-0.189(0.245)
	Connective tissue of the vaginal mucosa	-0.765(0.019)*	-0.126(0.241)	-0.236(0.243)	-0.110(0.152)

*p<0.05

## 4 Discussions

Neurophysiological and pathological studies have confirmed that POP and SUI patients have nerve damage that governs pelvic floor [9]. The immunohistochemical study of pelvic floor muscles had shown that female patients with POP or SUI have muscle fiber damage [10]. Changes in the pelvic floor muscles cause changes in their dominating muscles, which provides a morphological basis for nerve damage that can cause damage to the pelvic floor muscles [3, 11, 12]. Therefore, the damage of nerves of the female genitourinary tract was an important reason for POP and SUI in females. Gregory and his colleges [13] included 23 women who had undergone initial vaginal delivery in their study. They found all of initial vaginal delivery women had pelvic floor muscle and nerve damage. Snooks et al. [14] found that the partial denervation of the pelvic floor muscle tissue and vaginal neurological disorders during childbirth are particularly evident in patients with POP and SUI through neuroelectrophysiological examination. Neurological disorders can lead to local muscle atrophy, thinning and reduction in tension. In the process of muscle denervation, small needle-like muscle fiber aggregation, muscle atrophy, type I muscle fiber hypertrophy, and a small amount of scattered necrotic fibers may be present in the renervation of homo fiber aggregation after muscle denervation. Delancey [15] proposed that the pelvic floor muscles were weak due to pelvic floor nerve damage, thereby leading to pelvic floor support and pressure conduction disorder, which participates in the occurrence of POP and SUI. Neuromuscular pathology studies have also found that severe damage to nerve fibers results in the denervation of the governed muscles, such as muscle atrophy, muscle fiber keratosis, and homo fiber aggregation in enzyme staining, which is the process of denervation of the adjacent axis after the denervation of the muscle occurs [16, 17, 18].

Patients with PFD not only have reduced distribution of pelvic floor muscles but also reduced distribution of vaginal mucosal nerves. Establishing an animal model of SUI after vaginal delivery showed that the ganglion cells in the posterior vaginal plexus were significantly reduced compared with the normal control group. Teng et al. [19] examined tissues from the urethral opening of the anterior vaginal tract and found a decrease in nerve endings in the superficial layer of the SUI and POP groups. In this study, the expression of vaginal mucosal nerve fibers (neurofilament) in the POP and SUI groups was lower than that in control group, thereby indicating that the development of PFD may be caused by decrease in nerve fiber. Our results indicated that NPY expression showed a decreasing trend in the round ligament, cardinal ligament of uterus, uterosacral ligament, and vaginal anterior wall. Therefore, the decrease in neurotransmitter, especially the decrease in NPY, may reflect the changes in pelvic microcirculation, and the changes of microcirculation lead to abnormalities in nutrients and structures of muscle and connective tissue [5], thereby causing connective tissue to weaken and dysfunction. A certain amount of neuropeptide is necessary to maintain the normal structure and function of the pelvic floor [20]. NPY content in patients with POP and SUI changed, thereby indicating that they may play an important role in SUI development.

The role of NPY in POP and SUI pathogenesis, for example, the mechanism of the expression, distribution, and role of neuropeptides in this process and whether some neuropeptides and their regulators can be used for the prevention and treatment of PFD was not completely clear. This was the focus and direction of the next step of research in the field of PFD. Performing a systematic and in-depth study on this topic will aid in understanding PFD pathogenesis and provide a valuable basis for the clinical treatment of PFD.

## 5 Conclusion

NPY expression was reduced in the round ligament, cardinal ligament of uterus, Uterosacral ligament, and vaginal anterior wall of the patients with POP and SUI indicating NPY was correlated with the development of female pelvic floor dysfunction. However, the sample size of the four groups was small with limited statistical power. The correlation between NPY and female pelvic floor dysfunction should be further investigated through large prospective clinical study.
